# Characteristics of pulmonary perfusion and ventilation in healthy adults: a prospective observational study with phase-resolved functional lung magnetic resonance imaging

**DOI:** 10.1186/s12880-026-02519-5

**Published:** 2026-06-22

**Authors:** Anqi Liu, Yifei Ni, Jianping Wang, Linfeng Xi, Haoyu Yang, Hongyi Wang, Jie Du, Ling Zhang, Jinzhu Dai, Ke Huang, Yanhong Ren, Shiyao Wang, Jingen Xia, Jing An, Robert Grimm, Andreas Voskrebenzev, Jens Vogel-Claussen, Min Liu

**Affiliations:** 1 Chinese Academy of Medical Sciences & Peking Union Medical College, Beijing, 100005 China; 2 Department of Radiology, China-Japan Friendship Hospital, No.2 Yinghua Dong Street, Hepingli, Chao Yang District, Beijing, 100029 China; 3 Capital Medical University, Beijing, 100069 China; 4 Department of Pulmonary and Critical Care Medicine, China-Japan Friendship Hospital, Beijing, 100029 China; 5 Peking University China-Japan Friendship School of Clinical Medicine, Beijing, 100191 China; 6 DL Department, Siemens Shenzhen Magnetic Resonance Ltd., Shenzhen, China; 7 Research & Clinical Translation, Magnetic Resonance, Siemens Healthineers AG, Erlangen, Germany; 8 Department of Diagnostic and Interventional Radiology, Charité Universitätsmedizin Berlin, Berlin, Germany

**Keywords:** Phase-resolved functional lung magnetic resonance imaging, Lung, Perfusion, Ventilation, Flow-volume loop, Breath

## Abstract

**Background:**

Phase-resolved functional lung magnetic resonance imaging (PREFUL-MRI) enables simultaneous, free-breathing, radiation-free assessment of regional lung perfusion and ventilation. This study aimed to provide preliminary reference data for lung perfusion and ventilation using PREFUL-MRI in healthy adults, and to characterize the physiological dependencies of these metrics on two breathing patterns, sex, and age.

**Methods:**

In this prospective observational study, 87 healthy adults underwent PREFUL-MRI at 1.5 T during both normal and deep-slow breathing. Perfusion- and ventilation-related metrics were quantified via an automated pipeline. Paired comparisons between breathing states were performed using Wilcoxon signed-rank tests; unpaired comparisons between sexes and age groups (< 45 vs. ≥45 years) used Mann–Whitney U tests, with Holm–Bonferroni and Benjamini–Hochberg false-discovery-rate corrections applied to control for multiple comparisons.

**Results:**

Mean perfusion (7.7% vs. 6.0%, Holm-Bonferroni adjusted *p* < 0.001) and ventilation defects (8.6% vs. 5.1%, Holm-Bonferroni adjusted *p* = 0.010) were decreased, and mean ventilation (15.8% vs. 48.3%, Holm-Bonferroni adjusted *p* < 0.001) and perfusion defects (1.9% vs. 7.9%, Holm-Bonferroni adjusted *p* = 0.005) increased during deep breathing compared with normal breathing. Twenty-eight participants had increased lung perfusion while 59 had reduced perfusion during deep breathing relative to normal breathing. During normal breathing, men exhibited higher mean ventilation than women (20.2% vs. 14.2%, Holm-Bonferroni adjusted *p* = 0.018). During deep breathing, men demonstrated higher total perfusion defect percentage and matched ventilation-perfusion defects than women (FDR adjusted q < 0.05). Total perfusion defect percentage was lower in participants aged ≥ 45 years than in those aged < 45 years (1.8% vs. 2.7%, FDR adjusted q = 0.036). Mean flow-volume loop correlations were similar between breathing patterns, sexes, and age groups after multiple comparison correction (*p* > 0.05).

**Conclusions:**

PREFUL-MRI can captures physiological variations related to breathing pattern, sex, and age in healthy adults. These findings provide a framework for distinguishing normal physiological heterogeneity from pathological change in clinical PREFUL-MRI interpretation.

**Clinical trial number:**

Not applicable.

**Supplementary Information:**

The online version contains supplementary material available at https://doi.org/10.1186/s12880-026-02519-5.

## Introduction

Lung perfusion and ventilation are essential physiological function and are usually evaluated under illness conditions. Imaging techniques such as ventilation/perfusion single photon emission computed tomography and planar scintigraphy have been used to evaluate these functions in disease states and healthy individuals [1–3], dual-energy computed tomography [4] have been used to evaluate these functions in lung diseases such as pulmonary vascular disease and chronic obstructive pulmonary disease (COPD) [5]. However, their use in healthy individuals is uncommon owing to the radiation exposure, thus, pulmonary perfusion and ventilation have rarely been directly visually analysed in healthy individuals, particularly regarding physiological alterations during normal and deep breathing.

Phase-resolved functional lung magnetic resonance imaging (PREFUL-MRI) overcomes these limitations by enabling simultaneous, free-breathing, non-contrast assessment of ventilation and perfusion without radiation exposure [5–8]. Previous studies have primarily focused on validating PREFUL-MRI against reference standards in disease populations such as COPD, chronic thromboembolic pulmonary hypertension (CTEPH), and cystic fibrosis demonstrating its sensitivity to pathological changes and its correlation with pulmonary function tests (PFTs) [5–12]. Notably, Glandorf et al. compared PREFUL-MRI perfusion and ventilation parameters between 1.5T and 3T in healthy volunteers, demonstrating technical reproducibility and field-strength effects, however, the study was limited to a single coronal slice, did not evaluate physiological variations under different breathing patterns, and did not establish comprehensive normative reference values stratified by sex and age [13]. Therefore, our primary hypothesis is that deep-slow breathing significantly increases mean ventilation and perfusion defects but decreases mean perfusion and ventilation defects compared to normal breathing. The primary objective of this study was to establish preliminary reference data for pulmonary perfusion and ventilation metrics of PREFUL-MRI in healthy adults. Secondary objectives include investigating breathing pattern-, sex- and age-related variations in these metrics.

## Methods

### Study design and participants

The study was conducted in accordance with the Declaration of Helsinki (as revised in 2013) and followed the STROBE (Strengthening the Reporting of Observational Studies in Epidemiology) reporting guideline. The study was approved by the Ethics Board of China-Japan Friendship Hospital (2022-KY-031), and all participants provided written informed consent. This was a single-center, prospective observational study conducted from May 2023 to May 2024. Participants were consecutively recruited from adults undergoing annual physical examinations at our hospital’s Health Management Center. All participants first completed pulmonary function tests (PFTs), electrocardiogram (ECG), and low-dose CT (LDCT) within 24 h as screening assessments to confirm healthy status. Those without any abnormal findings on these screening tests then underwent PREFUL-MRI within 48 h.

**Fig. 1:**
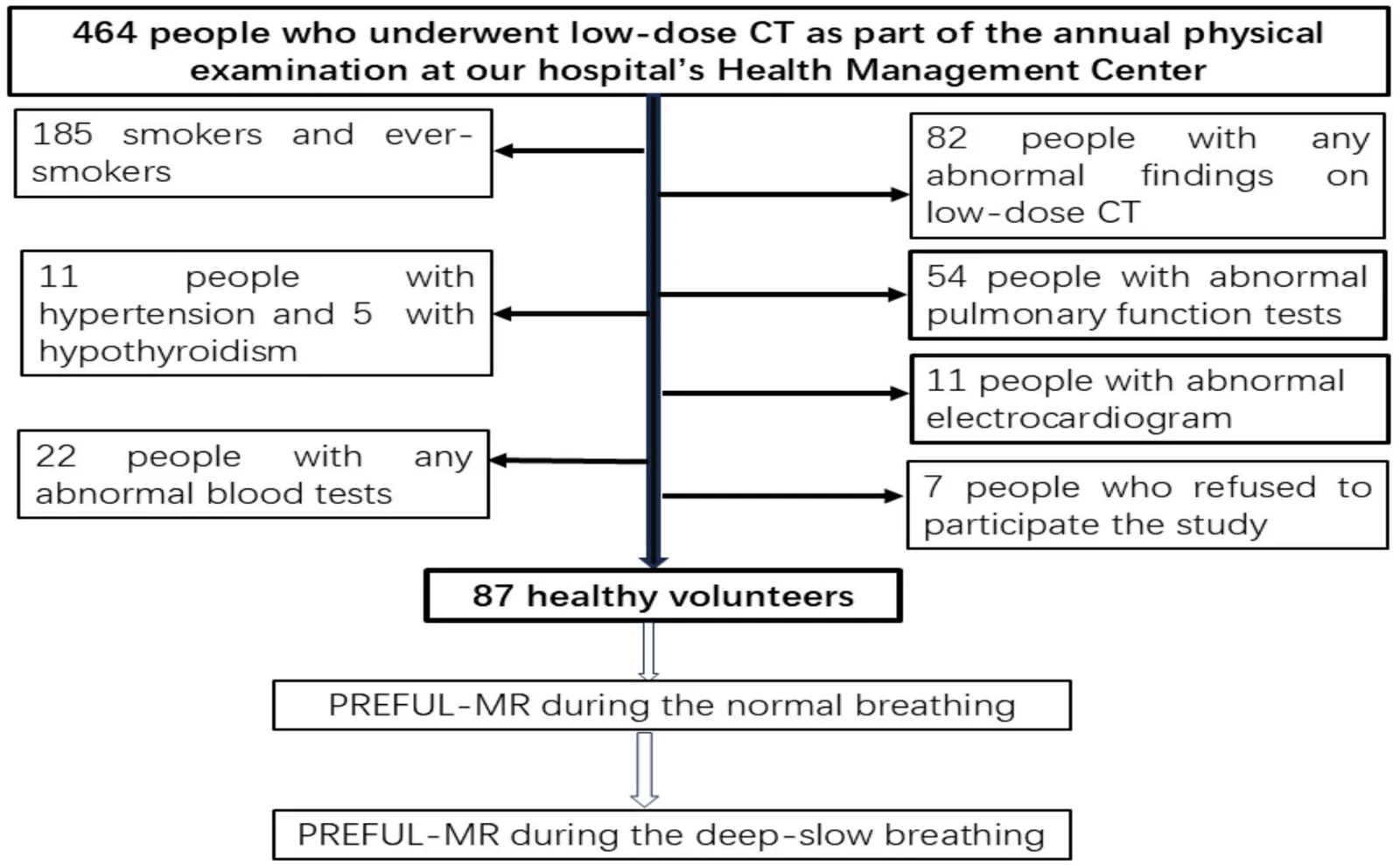
The flowchart detailing how participants were selected

Inclusion criteria were: (I).Adults (≥ 18 years of age) who underwent annual physical examination at our hospital’s Health Management Center between May 2023 and May 2024.(II)Completion of PFTs, ECG, and LDCT within 24 h as part of the physical examination. (III) No abnormal findings on PFTs, ECG, or LDCT.(IV) agree to undergo PREFUL-MRI within 48 h.(V) Ability to lie supine and follow standardized breathing instructions. Exclusion criteria were (I) former and current smokers; (II) individuals with any abnormal findings on PFTs, ECG, or LDCT; (III) individuals with history of any cardiopulmonary, systemic disease or malignancy; (III) individuals with history of coronary stent, pacemaker, aortic stent, or spinal surgery; and (IV) individuals who refused to undergo PREFUL-MRI.(V) Claustrophobia or any other contraindication to MRI. Figure 1 is the flowchart detailing how healthy participants were selected.

### Standardized breathing pattern instructions

Before MRI scan, all participants received standardized breathing instructions from a respiratory physician. For the normal breathing, participants were asked to breathe normally and quietly at their natural resting rate without deep inspiration or forced exhalation. For the deep‑slow breathing, participants were instructed to inhale deeply over approximately 3–4 s, then exhale slowly over approximately 4–5 s, aiming for a breathing frequency of 6–8 breaths per minute. Participants practiced both patterns in the supine position on a stretcher outside the scanner room for 2–3 min while the respiratory physician observed chest and abdominal wall motion. Correct performance was confirmed by the respiratory cushion signal placed on the upper right abdomen showing regular low‑amplitude excursions for normal breathing and high‑amplitude, slower excursions for the deep‑slow breathing.

### PREFUL-MRI scan

All participants underwent chest MRI on a 1.5Tesla MRI scanner (MAGNETOM Aera; Siemens Healthineers AG, Erlangen, Germany) equipped with an 18-channel phased-array body coil and a 12-channel spine coil. A transversal localiser was used to define the scan range, then PREFUL-MRI was conducted first during normal breathing, then during deep-slow breathing with a 2D fast low-angle shot gradient echo sequence (FLASH). The key scanning parameters included: Repetition Time(TR) = 1.86 ms, Echo Time(TE) = 0.74 ms, Flip Angle(FA) = 5°, Slice Thickness = 15 mm, Bandwidth = 1502 Hz/pixel, Matrix Size = 128 × 128, Field of View(FOV) = 500 × 500 mm, and Parallel Imaging Acceleration factor(GRAPPA) = 2. The PREFUL protocol acquired 5–8 coronal slices covering the whole lung from anterior to posterior. For each slice, 300 images were acquired over 59 s. The total PREFUL protocol duration ranged from 10 to 16 min depending on the number of slices.

### PREFUL postprocessing and analysis

PREFUL datasets were processed using MR Lung prototype v2.2.0 (Siemens Healthineers, Erlangen, Germany), including image pre-processing and registration, automatic lung segmentation, filtering, phase sorting and functional map generation as previously described [12, 14]. First step is image pre-processing and registration. Elastic registration was performed using a mid-ventilation image as reference. To achieve full saturation and ensure a steady state of the stationary tissue, the first 20 images of every PREFUL dataset were discarded. Second step is lung segmentation. Lung boundaries were semiautomatically segmented using a previously trained U-Net convolutional neural network(implemented in MR Lung prototype v2.2.0). Segmentation accuracy was verified by a chest radiologist with 12 years of experience in thoracic imaging (> 5000 chest MRI examinations, > 10,000 chest CT scans) and manually corrected when necessary. Segmentation accuracy was verified by a second independent chest radiologist with 22 years of experience in thoracic imaging. Third step is phase sorting and functional map generation. Images were automatically sorted based on ventilation and perfusion phases. The pulmonary perfusion phase was automatically selected from the virtual pulse wave, which was derived by temporally filtering the image time series (band‑pass filter: 0.5‑2.5 Hz, corresponding to heart rate 30‑150 bpm). Normalized perfusion (in arbitrary units, a.u.) was calculated voxel‑wise as the signal amplitude at the cardiac frequency divided by the median signal of full‑blood voxels (identified as voxels in the central slice with signal intensity > 90th percentile of the whole‑lung intensity). The ventilation phase was derived from the respiratory signal (0.15‑0.5 Hz), and ventilation maps were computed as the fractional change in signal intensity between end‑inspiration and end‑expiration. A perfusion and ventilation map and a regional flow-volume loop (FVL) map were analysed as described previously [6, 15]. For each lung voxel, the regional FVL was constructed by plotting the regional ventilation signal (serving as a surrogate for volume, analogous to lung volume in spirometry) against its first derivative over time, producing a loop morphology comparable to the global flow-volume loop obtained from spirometry. The FVL correlation was then computed as the Pearson correlation coefficient between each voxel-wise FVL and a reference FVL derived from the global ventilation signal of the entire lung. This metric reflects the degree to which regional ventilation dynamics mirror the overall, healthy lung ventilation pattern: values approaching 1.0 indicate highly regular and synchronized regional ventilation, whereas lower values suggest regional asynchrony, airflow limitation, or altered mechanical properties. The mean FVL correlation was averaged across all lung voxels for each participant. Finally, QDP maps, ventilation defect percentage (VDP) maps and perfusion/ventilation match (VQM) maps were automatically calculated. Supplemental Table 1 defines all PREFUL-MRI-related metrics for perfusion and ventilation.

### Pulmonary function assessment

All participants performed PFTs in accordance with the recommendations of the American Thoracic Society and European Respiratory Society [MasterScreen, Vyaire Medical GmbH] [16]. The measurements included the percentage of predicted forced vital capacity (FVC%), the percentage of forced expiratory volume in 1 s (FEV1%), the FEV1/FVC ratio, the percentage of predicted total lung capacity (TLC%), and the percentage of predicted diffusing capacity of the lungs for carbon monoxide corrected for measured haemoglobin (DLco%).

### Statistical analysis

Statistical analyses were conducted using SPSS version 26.0 (IBM Corp, Armonk, NY, USA) and MedCalc software for Windows version 20.0.22 (MedCalc Software Ltd, Ostend, Belgium). Data normality was assessed using the Shapiro–Wilk test prior to performing statistical tests. Data are reported as means ± standard deviation for normally distributed variables, and as medians with interquartile range (IQR) for non-normally distributed variables. Sex distribution and age group proportions were compared using chi-square tests or Fisher’s exact test as appropriate. As these comparisons involved repeated measurements from the same participants under two conditions (normal vs. deep breathing), paired-sample t-tests were used for normally distributed continuous variables, and Wilcoxon signed-rank tests were used for non-normally distributed variables. For comparisons between women and men, independent-sample t-tests were applied to normally distributed continuous variables, while Mann-Whitney U tests were used for non-normally distributed variables. For comparisons between participants aged < 45 years and ≥ 45 years, independent-sample t-tests were applied to normally distributed continuous variables, while Mann-Whitney U tests were used for non-normally distributed variables. To address multiple comparisons, we furtherly applied both Holm-Bonferroni sequential correction (controlling family-wise error rate) and Benjamini-Hochberg FDR correction (controlling false discovery rate at 5%) to each family of hypotheses. Given the absence of prior published effect sizes for PREFUL-MRI metric changes during deep-slow breathing in healthy adults, a formal a priori sample size calculation was not feasible at the study design stage. Post-hoc power analyses were performed for the primary endpoint comparisons (normal vs. deep breathing). A p-value < 0.05 was considered statistically significant.

## Results

### Participant characteristics

Eighty-seven healthy participants (47 men, mean age: 38.6 ± 12.3 years, range: 23–65 years) were enrolled in this study. Table 1 presents the demographics and clinical characteristics of all participants. Blood test results and PFTs were all within normal reference ranges, further confirming the healthy status of the cohort. As shown in Supplementary Table 2, there was no significant difference in age distribution or age group stratification between women and men (*p* > 0.05 for all comparisons), ensuring unbiased comparisons in subsequent sex-based analyses. Post-hoc power analyses show the achieved power exceeded 89% for all primary metrics and > 99% for three of four comparisons, indicating that the sample size of *n* = 87 was sufficient to detect the observed physiological differences (Supplementary Table 3).

**Table 1:** Participants’ demographics and clinical characteristics

Characteristic	Value
Total number	87
Women/men	40/47
Mean age (years)	38.6 ± 12.3 (23–65)
Aged < 45 years(n)	54
Aged ≥ 45 years(n)	33
Body mass index (kg/m^2^)	22.6 ± 5.1
Systolic blood pressure (mmHg)	112 ± 5.1
Diastolic blood pressure (mmHg)	70 ± 4.2
**Pulmonary function test**	
FVC% pre	105.7 ± 9.2
FEV1% pre	102.6 ± 8.8
FEV1/FVC%	83.1 ± 9.2
DLco% pre	98.7 ± 10.8
**Blood test**	
Red blood cells (×10^12^/L)	4.55 ± 0.31
White blood cells (×10^9^/L)	7.14 ± 1.23
Neutrophils (×10^9^/L)	4.28 ± 1.17
Lymphocytes (×10^9^/L)	2.01 ± 1.03
Haemoglobin (g/L)	141 ± 23
Platelets (×10^9^/L)	255 ± 83
Glycosylated haemoglobin (%)	4.8 ± 0.4
D-dimer(mg/L)	0.12(0.09,0.14)

### Perfusion and ventilation during normal and deep breathing

**Fig. 2:**
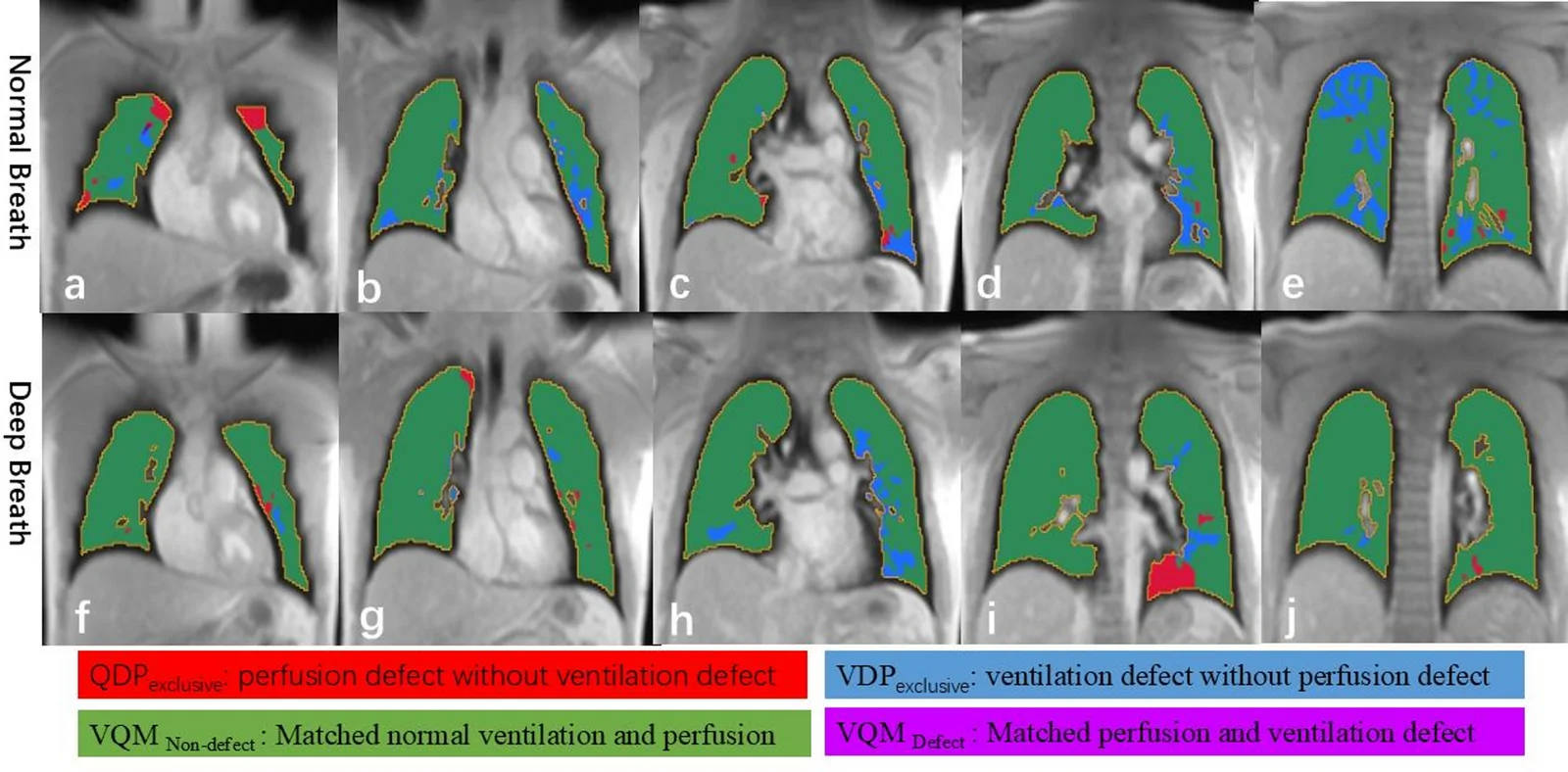
Whole-lung ventilation and perfusion maps acquired using PREFUL-MRI in a healthy 45-year-old man during normal breathing (Row 1, panels a–e, anterior to posterior) and deep-slow breathing (Row 2, panels f–j, anterior to posterior). Color coding: red, QDP_exclusive_ (perfusion defect without ventilation defect); blue, VDP_exclusive_ (ventilation defect without perfusion defect); green, VQM_non−defect_ (matched normal ventilation and perfusion); purple, VQM_defect_ (matched perfusion and ventilation defect). Abbreviations: PREFUL = Phase-resolved Functional Lung

**Fig. 3:**
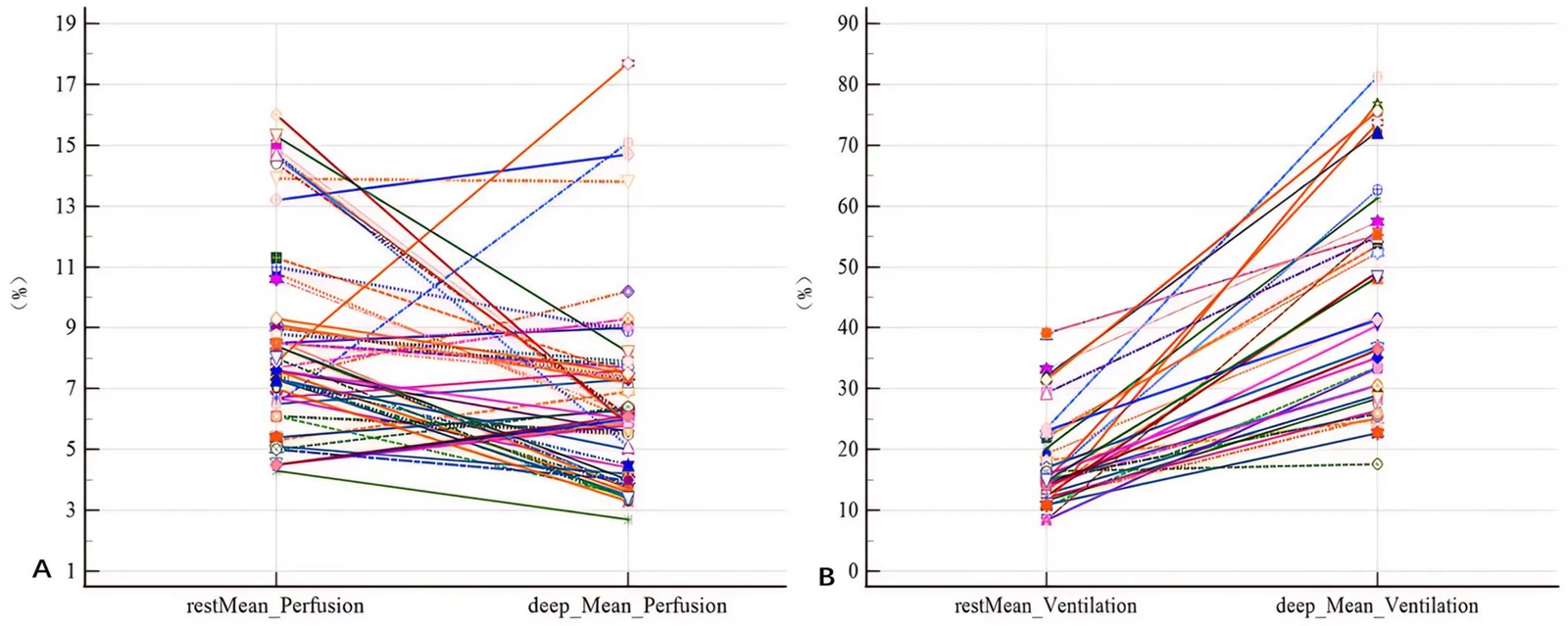
Changes in mean ventilation and perfusion between normal and deep-slow breathing in 87 healthy participants. (**A**) Individual mean perfusion values during normal and deep-slow breathing. (**B**) Individual mean ventilation values during normal and deep-slow breathing

Figure 2 illustrates the combined ventilation and perfusion maps of the whole lung during normal and deep breathing. During normal breathing, perfusion defects were observed primarily in the anterior lung apex, and ventilation defects were mainly distributed in the posterior part of the lung. During deep breathing, perfusion defects in the apex and ventilation defects in the posterior regions decreased. Figure 3A shows that mean perfusion varied among participants during deep breathing: 28 participants exhibited increased perfusion, while 59 showed decreased perfusion compared to the normal breathing. Figure 3B demonstrates that mean ventilation significantly increased during deep breathing in all 87 participants compared to normal breathing.

Table 2 shows mean Perfusion, ventilation defects without perfusion defects (VDP_exclusive_) and total ventilation defect percentage (VDP_total_) decreased, and mean ventilation, perfusion defects without ventilation defects (QDP_exclusive_), and total perfusion defect percentage (QDP_total_) increased during the deep breathing compared with those during the normal breathing. The matched perfusion and ventilation defect (VQM_defect_), the healthy matched perfusion and ventilation (VQM_non−defect_) and mean FVL correlation were similar between the normal and deep breathing. After both Holm-Bonferroni and FDR correction, 6 of nine comparisons still remain statistically significant. This indicated the increased ventilation, increased QDP, and decreased VDP during the deep breathing are robust to multiple comparison correction.

**Table 2:** Comparison of perfusion- and ventilation-related metrics during normal and deep breathing (with 95% CI for median differences)

Perfusion- and ventilation-related metrics	Normal breathing (*N* = 87)	Deep breathing (*N* = 87)	Median difference (95% CI)	Z-score	*p*	Holm-Bonferroni adjusted *p*	Benjamini-Hochberg (FDR)
Mean perfusion (%)	7.7 (6.5, 9.1)	6.0 (4.4, 7.7)	-1.5 (-2.2 to -0.8)	-4.367	< 0.001*	< 0.001*	0.001*
Mean ventilation (%)	15.3 (12.8, 29.3)	48.3 (33.4, 55.3)	25.4 (18.6 to 32.1)	8.104	< 0.001*	< 0.001*	0.001*
QDP_Exclusive_ (%)	1.9 (1.1, 4.2)	7.9 (1.9, 15.6)	4.6 (2.1 to 7.9)	6.112	0.001*	0.005*	0.003*
QDP_Total_ (%)	2.3 (1.2, 4.4)	8.1 (2.0, 17.1)	5.0 (2.4 to 8.5)	5.053	< 0.001*	< 0.001*	0.001*
VDP_Exclusive_ (%)	8.6 (4.6, 14.4)	5.1 (4.0, 8.0)	-2.8 (-4.6 to -1.1)	-3.067	0.002*	0.010*	0.004*
VDP_Total_ (%)	8.6 (4.9, 14.5)	5.8 (5.1, 8.0)	-1.9 (-3.7 to -0.3)	-2.052	0.012*	0.048*	0.018*
VQM_Defect_ (%)	0.1 (0, 0.2)	0.2 (0, 0.3)	0.0 (0.0 to 0.1)	1.818	0.131	0.393	0.148
VQM_Non−Defect_ (%)	86.4 (84.1, 91.4)	84.0 (78.9, 90.0)	-1.8 (-4.2 to 0.5)	-2.231	0.069	0.276	0.088
Mean FVL Correlation	0.96 (0.95, 0.99)	0.97 (0.97, 0.98)	0.00 (0.00 to 0.01)	0.192	0.848	0.848	0.848

Data are presented as median (interquartile range). Median differences and 95% confidence intervals (CI) were calculated using the Hodges-Lehmann estimator for paired differences. Positive median differences indicate an increase during deep breathing compared to normal breathing; negative values indicate a decrease. Holm-Bonferroni: Sequential Bonferroni correction controlling family-wise error rate at α = 0.05. Benjamini-Hochberg: Controls false discovery rate at 5%. Statistically significant after both corrections

### Perfusion- and ventilation-related metrics between women and men

Table 3 compares the perfusion- and ventilation-related metrics between women and men during the normal and deep breathing, revealing distinct patterns. During normal breathing, men exhibited higher mean ventilation than did women (20.2% vs. 14.2%, Holm-Bonferroni adjusted *p* = 0.018). After both Holm-Bonferroni and FDR correction, only mean ventilation during normal breathing remained statistically significant between sexes. During deep breathing, men demonstrated higher QDP_exclusive_, QDP_total_, and VQM_defect_ than did women (FDR adjusted q < 0.05 for all three; Holm-Bonferroni adjusted *p* < 0.05 for QDP_total_ and VQM_defect_). VQM_non−defect_ was lower in men (FDR adjusted q = 0.031) but did not survive Holm-Bonferroni correction (*p* = 0.085). Notably, mean perfusion and mean FVL correlation did not differ significantly between sexes under either breathing condition after correction.

**Table 3:** Comparison of perfusion- and ventilation-related metrics between women and men during normal and deep-slow breathing (with 95% CI for median differences)

Perfusion- and ventilation-related metrics	Women (*N* = 40)	Men (*N* = 47)	Median difference (95% CI)*	Z-score	*p*	Holm-Bonferroni adjusted *p*	Benjamini-Hochberg (FDR)
**Normal breathing**							
Mean perfusion (%)	7.45 (6.1, 8.5)	8.4 (6.7, 9.1)	0.6 (-0.2 to 1.4)	-1.26	0.322	1	0.483
Mean ventilation (%)	14.20 (12.27, 16.02)	20.20 (14.1, 31.5)	5.2 (2.0 to 8.9)	-3.12	0.002†	0.018†	0.018†
QDP_Exclusive_ (%)	2.30 (1.05, 4.20)	1.70 (0.95, 4.60)	-0.2 (-0.8 to 0.6)	0.66	0.507	1	0.55
QDP_Total_ (%)	2.50 (1.13, 4.40)	2.10 (1.15, 5.10)	-0.1 (-0.9 to 0.7)	0.66	0.507	1	0.55
VDP_Exclusive_ (%)	6.30 (2.5, 9.55)	9.50 (4.75, 17.65)	2.4 (0.3 to 5.2)	-2.29	0.022‡	0.154	0.066
VDP_Total_ (%)	6.40 (2.5, 9.70)	9.50 (5.0, 18.9)	2.5 (0.2 to 5.6)	-2.39	0.017‡	0.136	0.066
VQM_Defect_ (%)	0.10 (0, 0.2)	0.10 (0, 0.50)	0.0 (0.0 to 0.1)	-1.23	0.219	1	0.394
VQ_~ Non−Defect_ (%)	89.95 (84.50, 95.2)	84.8 (79.4, 90.4)	-3.5 (-7.1 to 0.1)	1.56	0.12	0.27	0.27
Mean FVL correlation	0.97 (0.95, 0.98)	0.97 (0.96, 0.98)	0.00 (-0.01 to 0.01)	-0.6	0.55	1	0.55
**Deep-slow breathing**							
Mean perfusion (%)	6.20 (5.6, 9.0)	5.90 (3.7, 7.7)	-0.3 (-1.4 to 0.7)	0.36	0.36	1	0.482
Mean ventilation (%)	31.95 (25.75, 36.42)	55.0 (36.9, 67.45)	18.1 (9.8 to 27.5)	-3.89	< 0.001†	0.005†	0.005†
QDP_Exclusive_ (%)	3.7 (1.4, 8.2)	10.2 (2.65, 18.50)	4.5 (1.1 to 8.6)	-2.81	0.005†	0.035†	0.014†
QDP_Total_ (%)	3.8 (1.4, 10.05)	10.3 (2.70, 19.40)	4.6 (1.2 to 8.9)	-2.97	0.003†	0.024†	0.014†
VDP_Exclusive_ (%)	5.10 (3.67, 8.20)	5.10 (4.60, 7.30)	0.1 (-0.9 to 1.2)	0.29	0.773	1	0.773
VDP_Total_ (%)	5.20 (3.83, 8.20)	5.8 (5.10, 7.90)	0.3 (-0.2 to 1.0)	-0.89	0.375	1	0.482
VQM_Defect_ (%)	0.10 (0, 0.225)	0.2 (0.1, 0.3)	0.1 (0.0 to 0.2)	-2.75	0.006†	0.036†	0.014†
VQM_Non−Defect_ (%)	88.0 (83.18, 93.78)	82.1 (73.2, 90.0)	-4.9 (-9.1 to -0.8)	2.39	0.017‡	0.085	0.031†
Mean FVL correlation	0.98 (0.95, 0.99)	0.97 (0.97, 0.98)	-0.01 (-0.01 to 0.00)	-0.44	0.661	1	0.773

*Median differences (Hodges-Lehmann estimator) and 95% confidence intervals (CI) calculated for Mann-Whitney U comparisons. Positive differences indicate Women > Men; negative differences indicate Women < Men. †Statistically significant after both Holm-Bonferroni and Benjamini-Hochberg (FDR) corrections. ‡Nominally significant (*p* < 0.05) but did not survive both corrections; interpreted as exploratory

### Perfusion- and ventilation-related metrics between participants aged < 45 and ≥ 45 years

Table 4 compares the perfusion- and ventilation-related metrics between participants aged < 45 and ≥ 45 years during normal breathing. QDP_total_ was significantly decreased in participants aged ≥ 45 years compared with those aged < 45 years (Holm-Bonferroni adjusted *p* = 0.036; FDR adjusted q = 0.036). QDP_exclusive_ showed a nominal raw p-value of 0.029 but did not survive either Holm-Bonferroni correction (adjusted *p* = 0.232) or FDR (q = 0.131). All other metrics, including mean ventilation, mean perfusion, VDP_exclusive_, VDP_total_, VQM_defect_, VQM_non−defect_, and FVL correlation, were similar between the two age groups after multiple comparison correction.

**Table 4:** Comparison of perfusion- and ventilation-related metrics between adults aged < 45 and ≥ 45 years during normal breathing (with 95% CI for median differences)

Perfusion- and ventilation-related metrics	Aged < 45 years (*N* = 54)	Aged ≥ 45 years (*N* = 33)	Median difference (95% CI)*	Z-score	*p*	Holm-Bonferroni adjusted *p*	Benjamini-Hochberg (FDR)
Mean perfusion (%)	7.50 (6.70, 9.68)	8.50 (6.10, 8.55)	0.4 (-0.3 to 1.2)	-0.61	0.543	1	0.892
Mean ventilation (%)	15.0 (12.4, 23.2)	16.4 (14.8, 36.15)	2.8 (-0.4 to 7.9)	-1.71	0.087	0.609	0.261
QDP _Exclusive_ (%)	2.50 (1.33, 4.60)	1.60 (0.70, 2.50)	-0.7 (-1.4 to -0.1)	2.18	0.029‡	0.232	0.131
QDP _Total_ (%)	2.70 (1.9, 5.1)	1.80 (0.7, 2.5)	-0.8 (-1.5 to -0.3)	2.89	0.004†	0.036†	0.036†
VDP _Exclusive_ (%)	8.50 (4.6, 13.9)	9.50 (3.1, 17.65)	0.5 (-2.2 to 3.5)	-0.14	0.892	1	0.892
VDP _Total_ (%)	8.60 (4.98, 14.10)	9.50 (3.2, 18.9)	0.5 (-2.4 to 3.7)	-0.15	0.882	1	0.892
VQM _Defect_ (%)	0.10 (0, 0.7)	0.10 (0, 0.2)	0.0 (-0.1 to 0.0)	-1.09	0.274	1	0.617
VQM _Non−Defect_ (%)	88.35 (84.3, 91.90)	84.8 (79.4, 90.9)	-2.4 (-5.7 to 1.2)	0.23	0.82	1	0.892
Mean FVL correlation	0.97 (0.948, 0.973)	0.97 (0.951, 0.980)	0.00 (-0.01 to 0.01)	-0.5	0.615	0.892	0.89

*Median differences (Hodges-Lehmann estimator) and 95% confidence intervals (CI) calculated for Mann-Whitney U comparisons. Positive differences indicate Aged < 45 years > Aged ≥ 45 years; negative differences indicate Aged < 45 years < Aged ≥ 45 years. †Statistically significant after both Holm-Bonferroni and Benjamini-Hochberg (FDR) corrections. ‡Nominally significant (raw *p* < 0.05) but did not survive either correction; interpreted as exploratory

## Discussion

In this prospective single‑center study of 87 healthy adults, we show the initial descriptive data on PREFUL‑MRI‑derived perfusion and ventilation metrics during normal and deep‑slow breathing. There are several findings as follows: (I) For the primary endpoint, deep-slow breathing significantly increased mean ventilation and both total and exclusive perfusion defect percentages (QDP_total_ and QDP_exclusive_), while significantly decreasing mean perfusion and both total and exclusive ventilation defect percentages (VDP_total_ and VDP_exclusive_), after Holm–Bonferroni correction. (II) Mean ventilation was consistently higher in men than in women during both breathing states after multiple comparison correction, whereas mean perfusion did not differ significantly between sexes under either breathing condition. (III) Older adults (≥ 45 years) exhibited lower total perfusion defect percentages (QDP_total_) than younger adults (< 45 years), a finding robust to both Holm–Bonferroni and FDR correction. (IV) Mean flow-volume loop (FVL) correlation was similar between normal and deep breathing, between men and women, and between age groups after correction for multiple comparisons. These findings highlight the utility of PREFUL-MRI in capturing detailed physiological changes in lung function in healthy individuals.

Glandorf et al. previously examined PREFUL-MRI in healthy volunteers, their work focused on cross-scanner reproducibility using a single-slice acquisition at 1.5T and 3T [13]. By employing a whole-lung multi-slice protocol and a substantially larger cohort, our study provides clinically applicable normative values that account for breathing-pattern, sex, and age variations, thereby advancing the clinical utility of PREFUL-MRI beyond technical feasibility. PREFUL-MRI has been used to evaluate the severity of lung diseases such as COPD [5, 8, 17], cystic fibrosis [18–21], PE [14, 15], asthma [22], COVID [23–24] and chronic lung allograft dysfunction post-lung transplant [25–26]. In healthy individuals during normal breathing, QDP accounts for 1.1%–4.2% of the whole lung. The perfusion defect may indicate the lung’s physiological dead space. During the normal breathing, ventilation defects account for 4.6%–14.4% of the whole lung in our healthy volunteers. In the context of healthy individuals, the terms ‘perfusion defect’ and ‘ventilation defect’ as used in PREFUL-MRI refer to regions of relatively reduced signal compared to the whole-lung average. These do not indicate pathological lesions (e.g., pulmonary embolism or COPD). Instead, they reflect physiological heterogeneity including gravitational gradients, regional differences in alveolar recruitment, functional dead space, and slow-filling lung units. Thus, in healthy adults, QDP and VDP represent recruitable functional reserve rather than true defects. We have retained the conventional PREFUL-MRI terminology (QDP, VDP) for consistency with the existing literature, but emphasize that these metrics should be interpreted as indices of physiological variation in healthy populations.

Evaluating QDP and VDP in healthy adult provides a critical baseline for understanding and diagnosing respiratory diseases. Establishing these normative values allows clinicians and researchers to better identify and quantify abnormalities in patients with illnesses, thereby improving diagnostic accuracy and treatment strategies. During deep breathing, mean ventilation was notably increased, and VDP was correspondingly decreased, compared with that during normal breathing. During the deep inhalation, the diaphragm descends significantly, and the external intercostal muscles contract to elevate the ribs and expand the thoracic cavity. Consequently, the lung volume increases considerably, and the negative pressure in the thoracic cavity becomes very pronounced. This forces more air into the lungs, thereby enhancing lung ventilation. Additionally, during the deep inhalation, the alveoli and bronchi expand further and more extensively, allowing air to enter more alveolar units, resulting in increased lung ventilation and fewer ventilation defects. Moreover, we found a decrease in perfusion during the deep breathing. Lung perfusion is primarily influenced by intrathoracic pressure. During deep breathing, downward movement of the diaphragm and outward expansion of the ribs significantly increase the intrathoracic space, thus markedly reducing the intrathoracic negative pressure. The increased intrathoracic space places more external pressure on pulmonary vessels, compressing them to some extent. The capillaries may experience traction and compression from the surrounding lung tissue during deep inhalation, leading to increased pulmonary vascular resistance and a reduction and redistribution of the overall pulmonary blood flow. However, the enhanced negative pressure during deep inhalation helps improve venous return to the right heart, which positively affects cardiac output by increasing the volume of blood pumped into the pulmonary circulation. The changes in intrathoracic pressure and venous return during deep inhalation ultimately affect total blood flow in the pulmonary circulation. Understanding these physiological mechanisms enables better interpreting the variations in pulmonary perfusion during different breathing patterns and assessing their implications for respiratory health and diseases.

Individual assessments identified two distinct pulmonary perfusion patterns during the deep-slow breathing: 59 participants exhibited decreased perfusion, whereas 28 showed increased perfusion compared with normal breathing. We hypothesize that this inter-individual variability could reflect differences in breathing mechanics (e.g., diaphragmatic versus thoracic dominance), which may influence venous return and pulmonary vascular resistance via differential intrathoracic pressure changes. However, we did not directly measure diaphragmatic excursion, chest wall motion, or cardiac output. Therefore, this interpretation remains speculative and hypothesis-generating, requiring future studies with concurrent respiratory motion tracking and hemodynamic monitoring.

Our results revealed sex-related differences in ventilation and perfusion metrics. Men showed higher ventilation than women during both breathing states, an effect that was robust to multiple comparison correction during normal breathing and deep breathing. The novel finding that men show higher QDP_total_ and VQM_defect_ during deep breathing was also robust under FDR correction. However, the sex difference in VDP during normal breathing and VQM_non−defect_ during deep breathing did not survive stringent Holm-Bonferroni correction and should be considered exploratory findings requiring validation in larger cohorts.

Younger healthy adults exhibit higher total perfusion defect percentages (QDP_total_) than older adults. This may reflect age-related changes in pulmonary vascular compliance, reduced physiological dead space, or altered distribution of ventilation-perfusion matching with aging. However, the nominal trend observed for QDP_exclusive_ (raw *p* = 0.029) did not survive correction and should be interpreted cautiously.These findings require validation in larger cohorts specifically powered for age-stratified analyses.

In the PREFUL analysis, the regional FVL illustrated regional ventilation (RV, serving as a surrogate for volume in spirometry)versus changes in RV ༈i.e., the first derivative of RV༉, analogous to FVLs in spirometry. The FVL correlation quantifies how closely the regional flow-volume trajectory matches the global reference loop, thereby serving as a marker of regional ventilation homogeneity and temporal synchrony throughout the respiratory cycle. In this study, the mean FVL correlation among healthy individuals ranged from 0.95 to 0.99, which was consistent between tidal and deep breathing, between men and women, and between those aged < 45 and ≥ 45 years. This near-unity correlation indicates that, in healthy lungs, regional ventilation dynamics are highly synchronized with global breathing patterns, with minimal regional asynchrony or airflow heterogeneity. Consequently, the mean FVL correlation appears to be a robust marker for assessing normal lung ventilation regularity, and future studies should evaluate whether its reduction correlates with obstructive or restrictive disease phenotypes. These findings highlight the utility of PREFUL-MRI in capturing detailed physiological changes in lung function in healthy individuals.

Moreover, the observation that ventilation defects decrease from 8.6% to 5.1% during deep breathing indicates that these regions retain ventilatory capacity but are not recruited during quiet breathing, a physiological economy of effort rather than a pathological impairment or defect. Similarly, the redistribution of perfusion defects suggests perfusion reserve that varies with hemodynamic conditions. So, we propose that in healthy individuals, VDP and QDP during normal breathing more accurately reflect functional heterogeneity or recruitable reserve capacity rather than true defects. This aligns with the concept of ‘silent spaces’ in respiratory physiology, lung units that remain unventilated at rest but contribute during exercise or forced maneuvers.

### Limitations

There are several limitations of this study that need to be addressed. First, the number of healthy participants was relatively small and the healthy population definition is highly selective, therefore, the descriptive values reported herein should be considered exploratory reference ranges from a well-characterized healthy cohort rather than true normative values for the general population. Multi-center studies with larger sample sizes and broader age coverage are necessary to establish genuine reference standards. Second, the large number of subgroup comparisons performed (normal vs. deep breathing, men vs. women, age < 45 vs. ≥45 years) inherently increases the risk of type I errors (false-positive findings). Although we applied both Holm-Bonferroni sequential correction and Benjamini-Hochberg false discovery rate (FDR) correction, and explicitly labeled findings that survived only one correction method as exploratory, some nominally significant associations may still represent chance findings. Therefore, all results, particularly those that did not survive both correction methods, should be interpreted as hypothesis-generating and require validation in independent, larger cohorts. Third, we did not perform concurrent spirometry or respiratory inductive plethysmography during MRI acquisition. Therefore, we cannot objectively quantify tidal volume, inspiratory capacity, or minute ventilation, nor can we exclude inter-individual variability in the actual depth of inspiration achieved during the deep-slow breathing task. The true physiological difference between normal and deep breathing cannot be objectively verified from our data. Future studies should incorporate MRI-compatible spirometry or thoraco-abdominal compartment monitoring to validate that the observed changes in PREFUL-MRI metrics are strictly volume-dependent and to standardize breathing maneuvers across participants. Fourth, we did not measure diaphragmatic excursion, chest wall compartment motion, or cardiac output. Consequently, our speculation regarding diaphragmatic versus thoracic breathing effects on perfusion is based on physiological reasoning rather than direct empirical evidence and should be regarded as hypothesis-generating only. Fifth, we assessed perfusion and ventilation only in the supine position. In the supine position, the gravitational gradient between lung apex and base is attenuated compared with the upright posture, potentially altering the regional distribution of perfusion and ventilation defects [27, 28]. Therefore, the values reported here should be interpreted as specific to the supine physiological state. Sixth, although we have retained the terms “perfusion defect” and “ventilation defect” for consistency with the PREFUL-MRI literature, we have clarified that in healthy individuals these represent physiological heterogeneity, not pathological defects. Finally, the lack of a formal a priori sample size calculation, our study may have been underpowered for some secondary subgroup comparisons. so, null findings should be interpreted cautiously, and the exploratory nature of the study is emphasized.

## Conclusion

This study reports preliminary reference ranges of PREFUL-MRI metrics in healthy adults, revealing that deep breathing paradoxically increases ventilation while decreasing global perfusion and increasing perfusion defect burden. We identify sex-specific physiological patterns and demonstrate that younger adults display higher perfusion defect than older adults, reflecting functional reserve rather than pathology. Clinically, these findings offer an initial framework for distinguishing normal physiological heterogeneity from disease-related abnormalities in PREFUL-MRI interpretation, pending validation in larger multi-center studies. They challenge conventional assumptions about ventilation-perfusion coupling and underscore the importance of considering breathing pattern, sex, and age when assessing pulmonary function with PREFUL-MRI.

## Supplementary Information

Below is the link to the electronic supplementary material.

Supplementary Material 1

## Data Availability

No datasets were generated or analysed during the current study.

## Abbreviations

PREFUL MRI: Phase-Resolved Functional Lung Magnetic Resonance Imaging
COPD: Chronic obstructive pulmonary disease
CTEPH: Chronic thromboembolic pulmonary hypertension
PFT: Pulmonary function tests
ECG: Electrocardiogram
LDCT: Low-dose CT
QDP: Perfusion defect percentage
VDP: Ventilation defect percentage
FVL: Flow-volume loop
FVC%: The percentage of predicted forced vital capacity
FEV1%: The percentage of forced expiratory volume in 1 s
TLC%: The percentage of predicted total lung capacity
DLco%: Carbon monoxide corrected for measured haemoglobin
IQR: Interquartile range
FDR: False discovery rate
VDP_exclusive_: Ventilation defects without perfusion defects
VDP_total_: Total ventilation defect percentage
QDP_exclusive_: Perfusion defects without ventilation defects
QDP_total_: Total perfusion defect percentage
VQM_defect_: The matched perfusion and ventilation defect
VQM_non−defect_: The healthy matched perfusion and ventilation
