# Supplementary material for: Characteristics of pulmonary perfusion and ventilation in healthy adults: a prospective observational study with phase-resolved functional lung magnetic resonance imaging
Source: BMC Med Imaging. 2026 Jun 22;26:437. doi: 10.1186/s12880-026-02519-5 (PMC13540945; doi:10.1186/s12880-026-02519-5)
Supplement: Supplementary file 1 [file 12880_2026_2519_MOESM1_ESM.docx]

**Supplementary Table 1. Definitions of PREFUL MRI parameters**

| **Parameter, units** | **Definitions** |
| --- | --- |
| Mean perfusion, % | Perfusion was normalized in reference to a full-blood signal region, derived as follows: (1) determine the parenchyma cardiac cycle phase through histogram analysis of the signal in the lung parenchyma, calculated as the time point in the reconstructed cardiac cycle when most of the lung parenchyma voxels reach their maximal value; (2) locate the full-blood signal region for normalization as the maximal intensity projection value in the lungs that which may to reflect the aorta or other available large vessel; and (3) divide the map of the reconstructed cardiac cycle signal at the parenchyma phase by the maximal value in the normalization region. |
| Mean ventilation, % | Fractional ventilation of the whole respiratory cycle calculated as: $\frac{S_{Mid}}{S_{Insp}}-\frac{S_{Mid}}{S_{Exp}}$with S the signal value at end-inspiration (*Insp*), end-expiration (*Exp*) and middle position (*Mid*) |
| VDP_total_, % | Percentage of areas identified as ventilation defects in total |
| VDP_exclusive_, % | Percentage of areas with ventilation defects but without perfusion defects |
| QDP_total_, % | Percentage of areas identified as perfusion defect in total |
| QDP_exclusive_, % | Percentage of areas with perfusion defects but without ventilation defects |
| VQM_defect_, % | Percentage of areas with concurrent perfusion and ventilation defects |
| VQM_non-defect_, % | Percentage of areas without perfusion defects or ventilation defects |

**Supplementary Table 2. Demographic and pulmonary function tests between male and female participants**

| **Characteristic** | **Women (n=40)** | **Men (n=47)** | **p-value** |
| --- | --- | --- | --- |
| Age (years) | 38.2 ± 11.8 | 38.9 ± 12.7 | 0.784† |
| Aged <45 years | 26 (65.0%) | 28 (59.6%) | 0.598‡ |
| Aged ≥45 years | 14 (35.0%) | 19 (40.4%) |  |
| Body mass index (kg/m²) | 21.8 ± 4.2 | 23.2 ± 5.7 | 0.203† |
| Pulmonary Function Tests |  |  |  |
| FVC% pre | 103.1 ± 8.5 | 107.9 ± 9.5 | 0.014*† |
| FEV1% pre | 101.4 ± 8.2 | 103.6 ± 9.2 | 0.246† |
| FEV1/FVC% | 85.2 ± 8.1 | 81.3 ± 9.8 | 0.048*† |
| DLco% pre | 96.3 ± 9.8 | 100.7 ± 11.2 | 0.056† |

†Independent t-test or Mann-Whitney U test; ‡Chi-square test. *p<0.05

**Supplementary Table 3. Post-hoc power analysis for the primary endpoint**

| **Primary endpoint comparison (normal vs. deep)** | **Observed Effect Size**  **(Cohen’s d)** | **Post-hoc Power**  **(α=0.05, two-tailed)** |
| --- | --- | --- |
| Mean perfusion | 0.47 (medium-large) | >99% |
| Mean ventilation | 0.87 (very large) | >99% |
| QDP_total_ | 0.54 (large) | >99% |
| VDP_total_ | 0.33 (medium) | 89% |
